# Research on Formation Conditions of the Ultrafine-Grained Structure of the Cylindrical Parts Manufactured by Power Spinning Based on Small Strains

**DOI:** 10.3390/ma11101891

**Published:** 2018-10-02

**Authors:** Gangfeng Xiao, Qinxiang Xia, Xiuquan Cheng, Weiping Chen

**Affiliations:** 1 Guangdong Provincial Key Laboratory of Precision Equipment and Manufacturing Technology, School of Mechanical and Automotive Engineering, South China University of Technology, Guangzhou 510640, China; xiaogf@scut.edu.cn (G.X.); mewpchen@scut.edu.cn (W.C.); 2 Department of Aircraft Maintenance Engineering, Guangzhou Civil Aviation College, Guangzhou 510403, China

**Keywords:** power spinning, ultrafine-grained structure, formation conditions, small strains, mechanical property

## Abstract

Two different methods, power spinning and annealing (PSA), quenching and power spinning followed by annealing (QPSA), for manufacturing the cylindrical parts with ultrafine-grained (UFG) structure were reviewed, the dislocation density and microstructural evolution during the two different processes of PSA and QPSA were further studied. The results show that the required strains for obtaining the UFG structure by power spinning is only 0.92 when the initial microstructure of the material is in the phase of lath martensite. The dislocation density and storage energy are increased to 10 times that of the blank after quenching and power spinning and decreased to the level of the blank after recrystallization annealing. Microstructures with fine grain size after quenching, storage energy of 1.8 × 10^5^ kJ/m^3^ obtained after power spinning and second phase particle with nano-scale precipitated during annealing are the necessary formation conditions for manufacturing the cylindrical parts with UFG structure based on small strains. Compared with the original tubular blank, the mechanical properties of the spun parts with UFG structure improves significantly. The tensile strength and hardness of the spun parts manufactured by QPSA method is 815 MPa and 305 HV, respectively, and the elongation is 17.5%.

## 1. Introduction

Grain refinement is one of the most important ways of increasing the strength and plasticity of materials simultaneously [1]. The materials will exhibit excellent mechanical properties when the grain size is refined to ultrafine scale (grain size ranged from 100 nm to 1000 nm) [2]. Severe plastic deformation (SPD) is the most common method for manufacturing the materials with ultrafine grained-structure (UFG), such as equal channel angular pressing (ECAP) [3], high pressure torsion (HPT) [4], multiple forging (MF) [5], accumulative roll bonding (ARB) [6] and so forth. However, large plastic strains are required to manufacture the materials with UFG structure by SPD processes, which is not benefit for the industrial applications [7].

Metal spinning is one of the near net-shape forming technologies to manufacture the thin walled-thickness and axisymmetrical parts [8]. It has inherent advantages, such as good dimensional accuracy and excellent mechanical properties [9,10]. Power spinning is a typical kind of spinning process, in which the plastic deformation is occurred and the wall thickness of the parts is reduced. It also can be used to manufacture the parts with a UFG structure [11,12]. What’s more, the method of manufacturing the materials with nano/ultrafine grained structures by combining the plastic deformation process and heat treatment is proposed to reduce the required strain [12,13]. Xia et al. [11,12] proposed a method for manufacturing the cylindrical parts with a UFG structure by combining power spinning process and heat treatment and the required plastic strains are reduced significantly.

The strain and forming method are playing an important role in the grain refinement. Umemoto summarized the SPD processes and pointed that the required strains of refining the grain to nano-scale need to reach 7–8 [7]. Xia et al. manufacture the cylindrical parts with a UFG structure by combining the spinning and heat treatment and the required strains is reduced to low levels [12]. For body centered cubic (BCC) metal, dislocation slip is the dominant plastic deformation mechanism. The dislocation density is closely related to the degree of grain refinement during manufacturing the materials with UFG structure, such as the SPD processes [14]. In deformed metals, the storage energy provided by dislocations is the driving force for recovery and recrystallization [15]. Therefore, the quantitative evaluation of the dislocation density is very important for studying the formation conditions of the UFG structure.

The methods of dislocation density measurement mainly include transmission electron microscopy (TEM) and X-ray diffraction (XRD) line profile analysis [16]. Only TEM can reveal the microstructural information in micro-region and the inhomogeneous of microstructure should be considered, whereas the XRD method can be used to measure the dislocation density in macro-regions [17]. Renzetti et al. [18] measured the dislocation of the 80% cold-rolled oxide dispersion strengthened (ODS) Eurofer steel annealed at different temperatures successfully using XRD. Lei et al. [19] studied the deformation, dislocation density and aging strengthening in Al-Cu-Mg alloy during artificial aging using XRD and established the function model of dislocation density and pre-deformation. Gubicza et al. [20] studied the dislocation density in a severely deformed copper specimen using the XRD method.

To explore the formation conditions of manufacturing cylindrical parts with a UFG structure, two different methods, power spinning and annealing (PSA), quenching and power spinning followed by annealing (QPSA), for manufacturing the cylindrical parts with UFG structure were reviewed and the dislocation density and microstructural evolution were studied with X-ray diffraction (XRD) and microstructural observations, respectively. The mechanical properties of the spun parts with a UFG structure are also analyzed.

## 2. Materials and Methods

### 2.1. Test Condition

**Figure 1:**
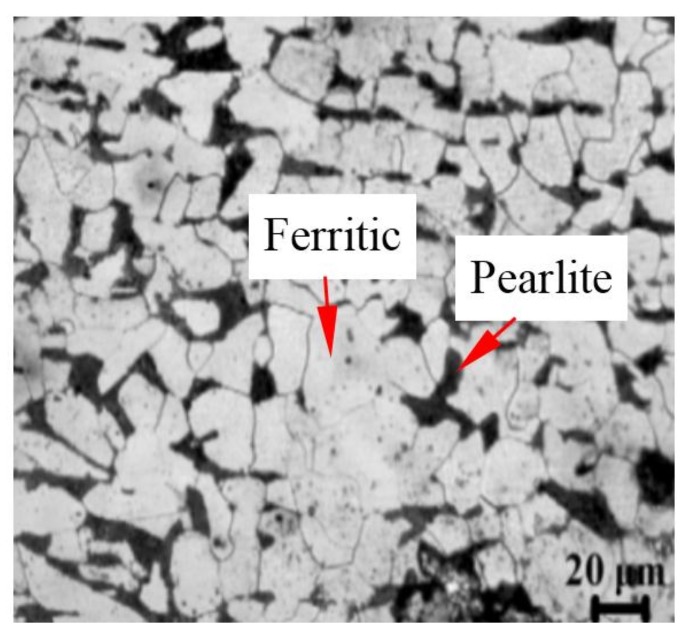
Initial microstructure of ASTM 1020.

The authors proposed two forming methods for manufacturing the cylindrical parts with UFG structure based on power spinning [11,12]. The cylindrical blank used for experiment is annealing seamless ASTM 1020, the material composition is shown in Table 1. The initial microstructure is ferritic and pearlite and the average size is 50 μm, as shown in Figure 1.

Method one: First, the 5-pass power spinning is performed with the total thinning ratio of 87%. Secondly, the spun parts are annealed at 480 °C for 60 min to form the equiaxed grains (PSA), the average grain size is refined to 600 nm [11].

Method two: First, the cylindrical blank is quenched at 910 °C for 10 min and then cooled quickly in the 5% NaCl solution to obtain the phase of lath martensite. Then the 3-pass power spinning process is performed with the total thinning ratio of 55%. Finally, the spun parts are annealed at 480 °C for 30 min to form the equiaxed grains (QPSA), the average grain size is refined to 160 nm [12].

**Figure 2:**
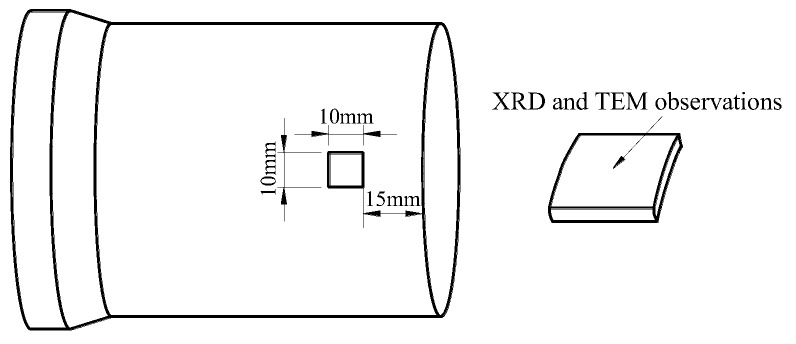
Sampling sketch of X-ray Diffraction (XRD) and Transmission Electron Microscopy (TEM) observations.

The XRD analysis is carried out in the workpieces during all stages of the process procedures with the D8 ADVANCE polycrystalline X-ray diffractometer (Bruker Company, Karlsruhe, Germany). The radiation wavelength is 0.15418 nm. The TEM observation is carried out with the JEM-2100 instrument (Japan electron optics laboratory Co., Ltd (JEOL), Tokyo, Japan). The samples for XRD and TEM observations are cut from the stable spinning area of the spun workpiece. The cutting distance from the opening area of the spun workpiece is about 15 mm and the surface of the sample is analyzed by XRD and TEM, as shown in Figure 2. The TEM samples are produced by mechanical polishing, followed by the electro-polishing based on the MTP-1A double jets instrument in the 4% HClO_4_ + 96%C_2_H_4_O_2_ at −40 °C and 75 V voltages.

### 2.2. Modified Williamdon-Hall Method Based on XRD

**Figure 3:**
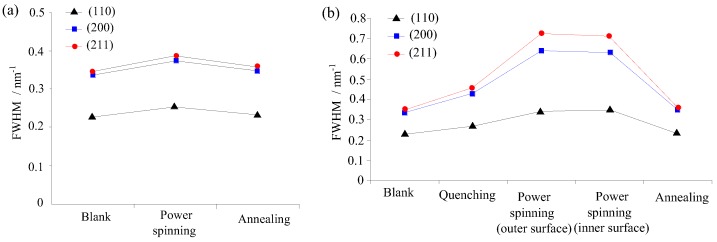
Full width at half maximum (FWHM) of diffraction peak of workpiece under different stages of manufacturing the cylindrical parts with ultrafine-grained (UFG) structure, (**a**) Power spinning and annealing (PSA), (**b**) Quenching and power spinning followed by annealing (QPSA).

The diffraction pattern of the sample is obtained by XRD analysis and imported to the Jade software (Jade5.0, MDI materials Data Inc, Livermore, CA, USA). Then the diffraction angle and the full width at half-maximum (FWHM) of diffraction peak are obtained by fitting the diffraction date of the samples. Three groups of samples are measured, which correspond to the three stages of the process procedures for manufacturing the parts with UFG by PSA (Figure 3a) and five groups of samples for that of PSA (Figure 3b). In addition, three samples were measured and averaged in each group to obtain the value of the FWHM.

The strain widening of the diffraction pattern is caused by dislocation piled-up in the crystal lattice [21]. Therefore, the full width at half-maximum (FWHM) of the diffraction peak can be evaluated by the Modified Williamson-Hall equation [17]:

(1) $$\;\Delta K=\frac{0.9}{D}+{\left(\frac{\pi M^{2}b^{2}}{2}\right)}^{1/2}\rho^{1/2}\left(K{\bar{C}}^{1/2}\right)+O\left(K^{2}\bar{C}\right)\;$$

where *K* = 2sinθ/λ, Δ*K* = cosθ[Δ(2θ)]/λ, θ is the diffraction angle, Δ(2θ) is the FWHM of the diffraction peak, λ is the wavelength of X-ray, *D* is the average grain size, $\bar{C}$ is the average constant factors of dislocations, *b* is the Burgers vector and the value is 0.25 nm; *ρ* is the dislocation density, *M* is the constant parameter, which is 1–2 for deformation body.

For cubic lattice, the average factors of dislocation for different diffraction vectors are defined as follows [22]:

(2) $$\;{\bar{C}}_{hkl}={\bar{C}}_{h00}\left(1-qH^{2}\right)\;$$

where ${\bar{C}}_{hkl}$ is a constant related to the elastic index of the material, *q* is the parameter related to the dislocation characteristics. *H*^2^ can be calculated by Equation (3) [20]:

*H*^2^ = (*h*^2^*k*^2^ + *h*^2^*l*^2^ + *k*^2^*l*^2^)/(*h*^2^ + *k*^2^ + *l*^2^)^2^ (3)

where *h*, *k*, *l* are the Miller indexes of the diffraction peak.

### 2.3. Calculation the Average Value of the Contrast Factors

For cubic lattice, the average value of the contrast factors can be calculated by Equation (4) [22]:

(4) $$\;{\bar{C}}_{h00}=a\left[1-exp(-A_{i}/b\right)]+cA_{i}+d\;$$

where *A*_i_ is the elastic anisotropy, *A**_i_*** = 2*C*_44_/(*C*_11_ − *C*_12_). *C*_11_, *C*_12_ and *C*_44_ are the three elastic constants, *a*, *b*, *c* and *d* are correlation coefficient. For the bcc lattice of Fe, *C*_11_ = 241 GPa, *C*_12_ = 146 GPa, *C*_44_ = 115 GPa [23]. Therefore, *A*_i_ = 2.42, *C*_12_/*C*_44_ = 1.27. For edges dislocation of bcc lattice, *a* = 1.6690, *b* = 21.124, *c* = 0, *d* = 0.0757.

The contrast factor is 0.300 for the screws dislocation and is 0.256 for edges dislocation. Therefore, the average value of the contrast factor is the average value of the screws dislocation and edges dislocation, ${\bar{C}}_{h00}=0.283$.

### 2.4. Calculation of the Parameter q

For cubic lattice, the parameter *q* of the dislocations can be calculated by Equation (5) [22]:

(5) $$\;q=a\left[1-exp(-A_{i}/b\right)]+cA_{i}+d\;$$

where the *A*_i_ is the elastic anisotropy, *a*, *b*, *c* and *d* are correlation coefficient.

For edges dislocation, *a* = 7.2361, *b* = 0.9285, *c* = 0.1359, *d* = −5.7484. For screws dislocation, *a* = 8.6590, *b* = 0.3730, *c* = 0.0424, *d* = −6.074 [22]. Therefore, the average value of the contrast factor is the average value of the screws dislocation and edges dislocation, *q* = 1.98.

### 2.5. Calculation of the Dislocation Density

**Figure 4:**
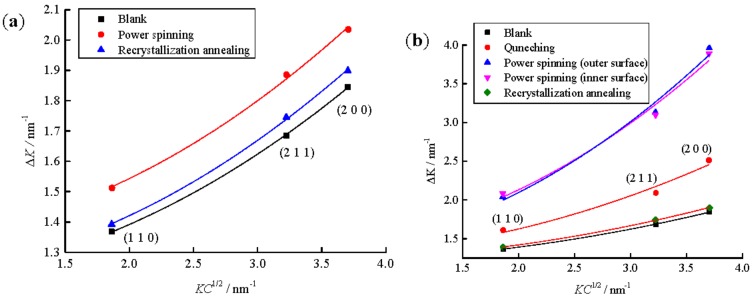
Modified Williamson–Hall plot of the FWHM for workpieces under different stages, (**a**) PSA, (**b**) QPSA.

The modified Williamson-Hall plots corresponding to the samples under different stages for manufacturing the cylindrical parts with UFG structure by PSA and QPSA are shown in Figure 4. It shows that all curves are fit well, which indicates that the selection of ${\bar{C}}_{h00}$ and *q* is reasonable. The slope of the fitted curve indicates the dislocation density given by *ρ* = 2*m*^2^/(*πM*^2^*b*^2^) with *m* is the slope of the linear fits on *K*^2^*C* variable [18].

## 3. Results and Discussion

### 3.1. Dislocation Density and Microstructural Evolution

**Figure 5:**
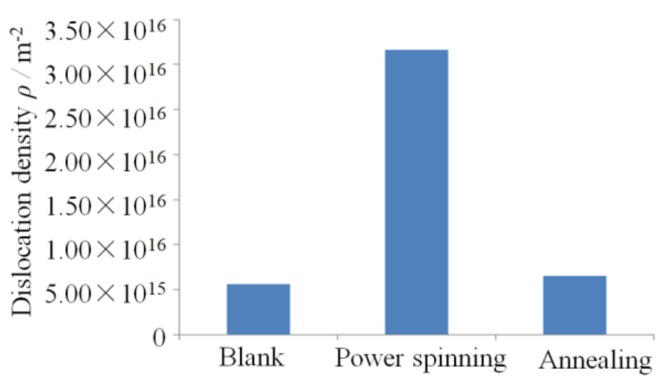
Dislocation density of workpieces under different stages of manufacturing the parts with UFG structure by PSA method.

**Figure 6:**
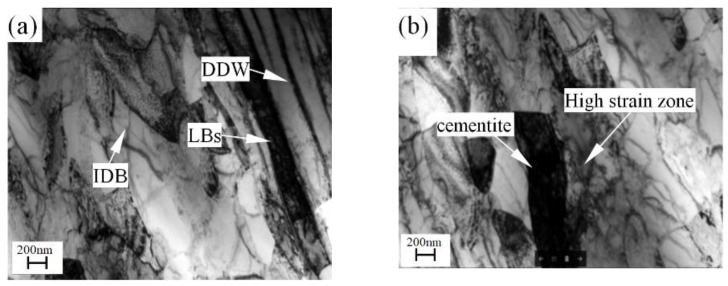
TEM micrographs of workpieces after power spinning of manufacturing the parts with UFG structure by PSA method, (**a**) Geometrically necessary boundaries (GNBs) and incidental dislocation boundaries (IDBs), (**b**) High strain zones.

**Figure 7:**
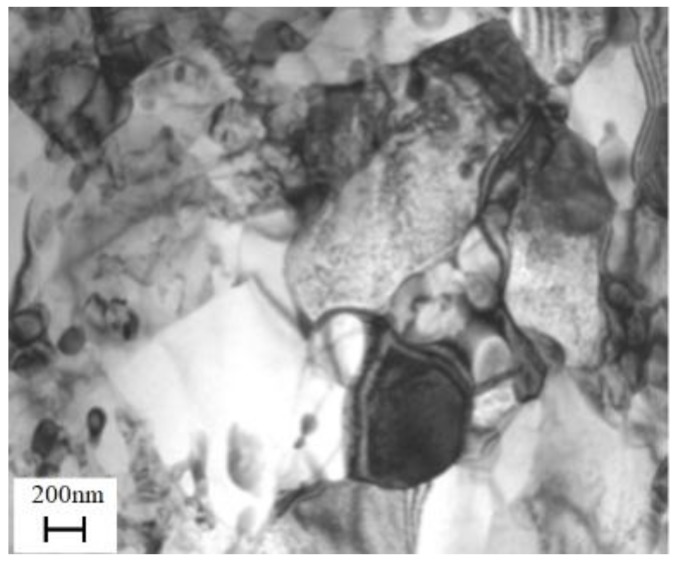
TEM micrographs of workpieces after recrystallization annealing of manufacturing the parts with UFG structure by PSA method.

Figure 5 shows the dislocation density of the workpieces under different process stages of manufacturing the cylindrical parts with UFG structure by PSA method. It shows that the dislocation density is increased from 5.6 × 10^15^ m^−2^ to 3.16 × 10^16^ m^−2^ after power spinning under 87% thinning ratio of wall thickness, the corresponding equivalent strain is 2.27. Figure 6 and Figure 7 show the TEM micrographs of workpieces under different process stages of manufacturing cylindrical parts with UFG structure by PSA method. They show that there are two kinds of dislocation boundaries existed in the microstructure after power spinning. One is geometrically necessary boundaries (GNBs), it mainly consists of dense dislocation walls (DDW) and lamellar boundaries (LBs), which is parallel to the deformation direction, as shown in Figure 6a. Meanwhile, the incidental dislocation boundaries (IDBs) are formed due to dislocation tangling, which is no macro orientation (Figure 6a). The cementite phase exists in the crystal lattice and the dislocation density around the cementite is much higher than that of other zones. Therefore, high strain zones are formed around the cementite, where will become a favorable nucleation zone during subsequent recrystallization annealing heat treatment (Figure 6b). The subgrain size is decreased and the low angle boundaries can transform to large angle boundaries with the increasing of the thinning ratio of wall thickness, which will result in the grain refinement [11].

However, the microstructure of the spun workpiece is non-equilibrium and large residual stress is existed in the microstructure. Moreover, the grain is elongated along the axial direction, even formed fibrous tissue. After recrystallization, the dislocation density is decreased close to that of the blank and the average grain size is refined to 600 nm (Figure 7). Therefore, compared with the SPD processes, the equiaxed UFG structure without distortion is formed and the required strain is reduced significantly.

**Figure 8:**
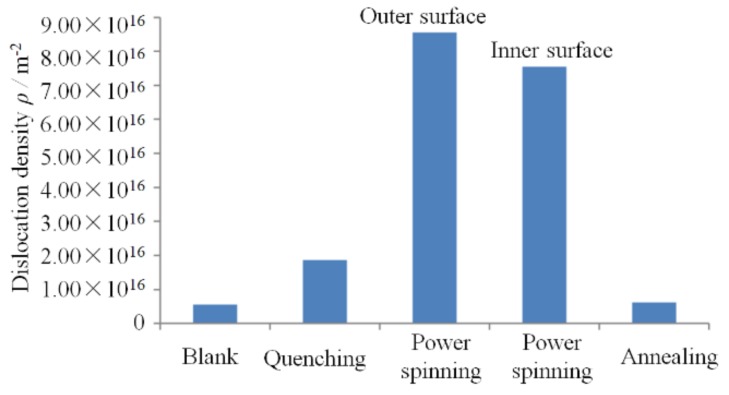
Dislocation density of workpieces under different stages of manufacturing the parts with UFG structure by QPSA method.

**Figure 9:**
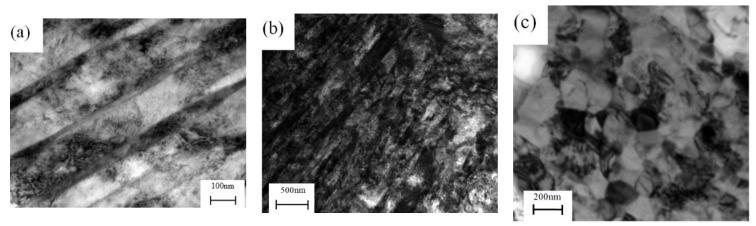
TEM micrographs of workpieces under different stages of manufacturing the parts with UFG structure by QPSA, (**a**) Quenching, (**b**) Power spinning (*Ψ*_t_ = 55%), (**c**) Recrystallization annealing.

Figure 8 and Figure 9 show the dislocation density and microstructural evolution of the workpieces under different process stages of manufacturing the cylindrical parts with a UFG structure by QPSA method, respectively. They show that the dislocation plays an important role in the grain refinement and the dislocation density in the crystal lattice are changed significantly during the forming processes. The dislocation is mainly concentrated in the grain boundaries (GBs), which is increased from 5.6 × 10^15^ m^−2^ to 1.87 × 10^16^ m^−2^ after quenching heat treatment. The microstructures of the ferrites and pearlites are transformed to lath martensites and a very high dislocation density is introduced, which leads to the formation of the intragranular structure such as dislocation cells and low misorientation angles. The lath martensite structure has three-level hierarchy: martensite lath, block and packet. The distribution of the dislocation in the martensite lath is inhomogeneous. The average thickness of the martensite lath is 170 nm. (Figure 9a).

The dislocation density is further increased rapidly and the distribution of dislocation is become more homogeneous after power spinning. The dislocation density of workpiece in the outer surface is reached to 8.55 × 10^16^ m^−2^. The ultrafine dislocation cells are formed in the interior of the deformed martensite lath (Figure 9b). Compared with the equilibrium microstructure (ferrite and pearlite) used in the QPSA method, the refining degree is improved and the dislocation density is increased significantly when the initial microstructure is the lath martensite during power spinning. This is because, on one hand, there are more grain and subgrain boundaries than equilibrium microstructure, such as the boundaries of martensite lath, block and packet and the dislocation can pile up near these boundaries during power spinning. On the other hand, the carbon atoms dissolved into the martensite matrix can segregate to form Cottrell atmosphere, which will hinder the dislocation movement and result in the dislocation tangling and the generation of dislocation cells. The dislocation density is larger and the distribution is more homogeneous than that of PSA method (Figure 9b). This provides more nucleation sites for the subsequent recrystallization annealing. Therefore, the microstructure of the workpiece can be changed significantly by quenching and power spinning and the dislocation density is increased to 10 times that of the blank.

As report by Xiao et al. [24], the percentage of deformation and grain refinement in the outer surface is far more than that of the inner surface when the microstructure of blank is pearlite and ferrite during mandrel spinning. This is because that the deformation first occurs in the outer surface and the friction between the workpiece and mandrel resists the metal flow on the inner surface of the workpiece. The resulting deformation amount in the outer surface is much larger than that of the inner surface. However, the dislocation density in the outer surface is slightly more than inner surface (7.55 × 10^16^ m^−2^) and deformation degree in the outer and inner surface is nearly the same after quenching and power spinning. This is because that the resistance effect of the friction on the metal flow for martensite structure is far less than that of the microstructure of pearlite and ferrite due to its high strength and hardness and the difference of the deformation amount between the inner and outer surface is decreased with the increasing of the thinning ratio of wall thickness. Therefore, the QPSA method is an effective method to realize the homogeneous grain refinement along the thickness direction.

The dislocation density of the workpiece is decreased to 6.24 × 10^15^ m^−2^ after recrystallization annealing at 480 °C for 30 min due to static recovery and recrystallization, which is close to the original blank. The ferrite grain with an average size of 160 nm is generated and the nano-cementite is precipitated in the ferrite matrix (Figure 9c). The cementite plays the pinning effect during recrystallization, which is beneficial for refining the grain size. Compared to the PSA method, the required plastic strain of refining the grain size to the UFG structure is further reduced to 0.92.

### 3.2. Formation Condition of the UFG Structure

The grain size after recrystallization is closely related to the microstructure, storage energy and second phase particle [25]. The degree of grain refinement of the parts obtained by QPSA method is larger than that of PSA method but the required plastic strain is much less than that of PSA method. The grain size is refined and large residual stress exists in the lattice of the grains after quenching and power spinning. Therefore, the recrystallization annealing should be carried out to obtain the refined equiaxed grains. The grain size after recrystallization annealing is related to the storage energy in the crystal lattice, which acts as the driving force. The relationship between the storage energy and the dislocation density can be calculated by Equation (6) [26,27]:

(6) $$\;E=\rho \frac{Gb^{2}}{2}\;$$

where *ρ* is the dislocation density, *G* is the shear modulus, the value is 79 GPa; *b* is Burgers vector.

**Figure 10:**
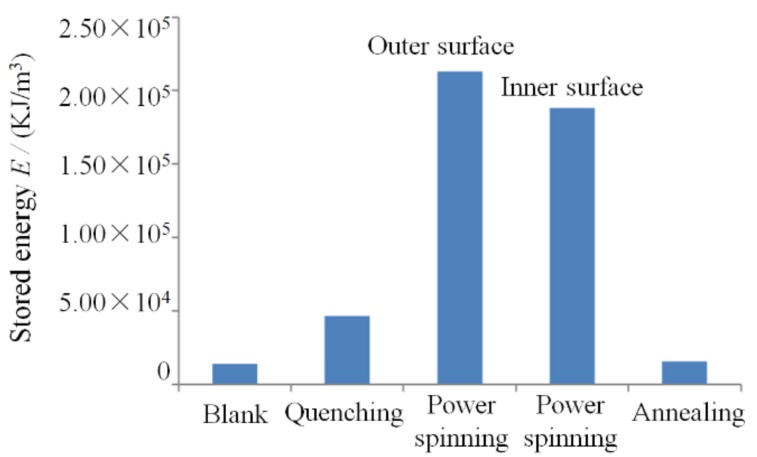
Stored energy of workpieces under different stages of manufacturing the parts with UFG structure by QPSA method.

Figure 10 shows the storage energy of the workpieces under different stages of manufacturing the parts using the QPSA method. It shows that the storage energy of workpieces after quenching and power spinning is increased from 1.38 × 10^4^ kJ/m^3^ to 2.13 × 10^5^ kJ/m^3^. Then it is decreased to 1.55 × 10^4^ kJ/m^3^ due to the energy consumption during recovery and recrystallization, which is closed to that of the blank.

The driving force of the recrystallization is the storage energy of the deformed metal, the value means the energy difference between the after and before recrystallization. Therefore, the difference of the storage energy is 1.73~1.98 × 10^5^ kJ/m^3^ for obtaining the ultrafine grains with the grain size of 160 nm.

The average grain size of the workpiece can be calculated by Equation (7) after recrystallization annealing for deformed metal [28]:

(7) $$\;d=K{\left(\frac{\dot{G}}{\dot{N}}\right)}^{1/4}\;$$

where $\dot{N}$ is nucleation rate, $\dot{G}$ is the grow rate of the grain, *K* is the proportionality constant.

The martensite lath with an average thickness of 87 nm is obtained and the dislocation cell with nanoscale in the lath interior is formed after quenching and power spinning. The zones, such as the boundaries of the packet, block and lath, have high energy, which is beneficial for the recrystallization nucleation. Therefore, the $\dot{N}$ is improved significantly due to the grain or subgrain boundaries and the dislocation cells existing in the grain interior. Secondly, the large storage energy exists in the lattice after quenching and power spinning, the value reaches 1.8 × 105 KJ/m^3^. The $\dot{N}$ and $\dot{G}$ are increased with the increasing storage energy but the increase rate of $\dot{N}$ is larger than that of $\dot{G}$. Therefore, the value of $\dot{G}/\dot{N}$ is decreased when the storage energy is improved. Moreover, the second phase particle at a nanoscale is generated during the recrystallization. In this study, the ASTM1020 steel was selected as the blank, the content of carbon is 0.2%, the cementite is precipitated in the grain interior and uniformly distributed in the ferrite matrix during the recrystallization. It inhibits the growth of ferrite grains and causes a decrease of $\dot{G}$.

### 3.3. Mechanical Properties of the Parts with UFG Structure

**Figure 11:**
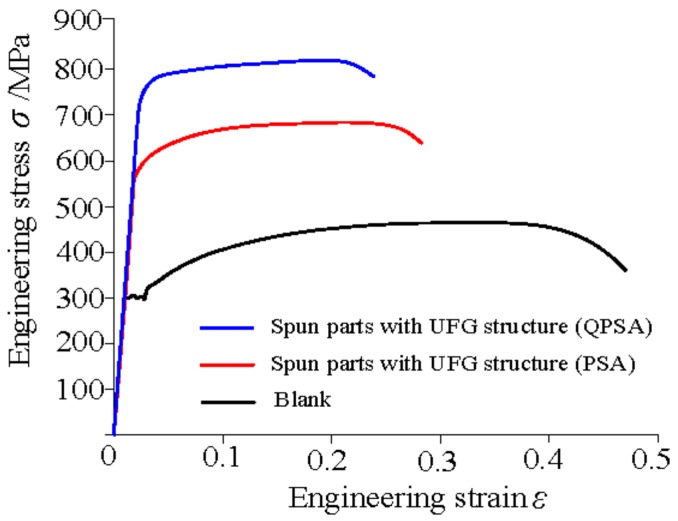
Engineering stress-stain curves of blank and spun parts with UFG structure.

**Figure 12:**
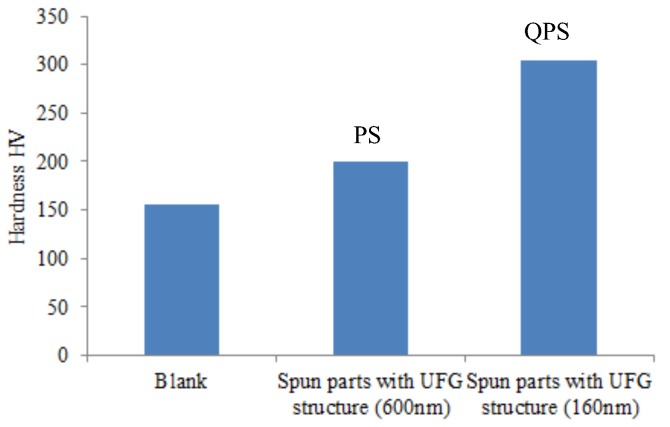
Hardness of the spun parts with UFG structure.

The superior properties of the parts can be obtained when the grain size is refined to the ultrafine scale [29]. The mechanical properties were obtained through the uniaxial tension test and engineering stress-stain curves of blank and spun parts with UFG structure are shown in Figure 11. Figure 12 shows the hardness of spun parts with a UFG structure, manufactured by PSA and QPSA. They show that the strength and hardness of the parts with a UFG structure are improved significantly. The tensile strength of the spun parts is increased from 465 MPa to 650 MPa and 815 MPa, manufactured by PSA and QPSA, respectively. The Hardness is increased from 155 HV to 200 HV and 305 HV, manufactured by PSA and QPSA, respectively. The strength and hardness of the materials is closely related to grain size and they are increased with the decreases of the grain size according to the Hall-patch relationship. However, the elongation decreases from 35% to 20% and 17.5% manufactured by PSA and QPSA, respectively. This is because the number of dislocations can pile-up in the grain interior is decreased significantly when the grain size decreases to the ultrafine scale. It leads to a decrease in strain hardening ability during plastic deformation and the plastic instability phenomenon occurs easily [2,30].

## 4. Conclusions

Two different methods, PSA and QPSA, for manufacturing the cylindrical parts with a UFG structure were reviewed. The formation conditions of the UFG structure of the cylindrical parts manufactured by power spinning were studied based on dislocation density measurement and microstructural evolution observation. The following conclusions can be drawn:

(1) The required plastic strains can be reduced significantly when manufacturing the parts with a UFG structure by combining power spinning and heat treatment technologies. The required plastic strains are only 2.27 and 0.92 of manufacturing the cylindrical parts with UFG structure by PSA and QPSA, respectively.
(2) The dislocation density and storage energy are increased to 10 times that of the blank after quenching and power spinning and decreased to the level of the blank after recrystallization annealing.
(3) The refining degree of the martensite structure during power spinning is much larger than that of the equilibrium structure. The ultrafine dislocation cells are formed in a martensite lath after power spinning and the UFG structure with an average grain size of 160 nm is generated after recrystallization annealing.
(4) The formation conditions for manufacturing the cylindrical parts with a UFG structure based on small strains are as follows: (1) Fine grains and subgrains size obtained during quenching; (2) The storage energy of the workpiece reaches 1.8 × 105 kJ/m3 after power spinning; and (3) Second phase particle with nano-scale is precipitated during recrystallization.
(5) The tensile strength and hardness of the ASTM 1020 spun parts manufactured by the QPSA method are 815 MPa and 305 HV respectively, while the elongation is decreased to 17.5%.

## Figures and Tables

**Table 1:** Material composition of ASTM 1020.

Materials	C/%	Si/%	Mn/%	P/%	S/%
ASTM 1020	0.20	0.21	0.51	0.015	0.008
